# Prelaminated Gracilis Flap with Buccal Mucosal Graft for Salvage of Devastated Urethra

**DOI:** 10.1155/2015/490518

**Published:** 2015-07-16

**Authors:** Dmitriy Nikolavsky

**Affiliations:** Department of Urology, SUNY Upstate Medical University, 750 E. Adams Street, Syracuse, NY 13210, USA

## Abstract

In patients with devastated bulbous urethra, that is, bulbar necrosis, failed fasciocutaneous repairs and “watering can perineum” repair options are limited by paucity of reliable local tissue suitable for reconstruction. In this case report we demonstrate a novel variation of a two-stage technique for reconstruction of a devastated bulbous urethra in a 57-year-old male who suffered penetrating trauma to his previously reconstructed urethra. Because of extensive loss of local tissue from the prior reconstruction and subsequent trauma and infection a 2-stage technique with use of gracilis was employed. This technique involved creation of two independently vascularized urethral hemi-plates prelaminated with buccal mucosa graft (BMG). In the first stage the dorsal plate was created by quilting buccal graft onto corpora cavernosa to create a temporary augmented perineal urethrostomy. In the same stage the future ventral neourethral plate was created by grafting another BMG onto the exposed distal gracilis muscle. Eight weeks later the two prelaminated plates were anastomosed by tunneling the gracilis-BMG composite into the perineum. At 8-month follow-up patient has normal voiding and continence. To our knowledge this is the first report of reconstructing an entire segment of devastated urethra in such a manner.

## 1. Introduction

In patients with devastated bulbous urethra, that is, spongionecrosis, failed fasciocutaneous repairs and a “watering can perineum” repair options are limited by paucity of reliable local tissue suitable for reconstruction. In this case report we demonstrate a novel two-stage technique for reconstruction of a devastated bulbous urethra. This 2-stage technique involves creation of two independently vascularized urethral hemi-plates prelaminated with buccal mucosa graft (BMG). In the first stage the dorsal plate is created by quilting BMG onto corpora cavernosa to create a temporary augmented perineal urethrostomy. In the same stage the future ventral neourethral plate is created by grafting another BMG onto the exposed distal gracilis muscle. During the second stage the two prelaminated plates are anastomosed by tunneling the gracilis into the perineum. To our knowledge this is the first report of reconstructing an entire segment of devastated urethra in such a manner.

## 2. Case Presentation

A 57-year-old male with a remote history of a two-stage fasciocutaneous urethroplasty presented with a new penetrating injury to his perineum after falling on a crowbar at work. He was reluctant to see a physician immediately and has delayed his visit by 3 months. By the time of his first visit he had a large perineal abscess and a fistula through which he was voiding since the injury. The patient was immediately admitted to the hospital and underwent incision, drainage, and conservative debridement of the perineal abscess and a suprapubic tube placement. On urethroscopy the urethral lumen could not be visualized beyond proximal pendulous urethra. The patient was managed with wet to dry dressing changes as outpatient until complete closure of the perineal wound.

**Figure 1:**
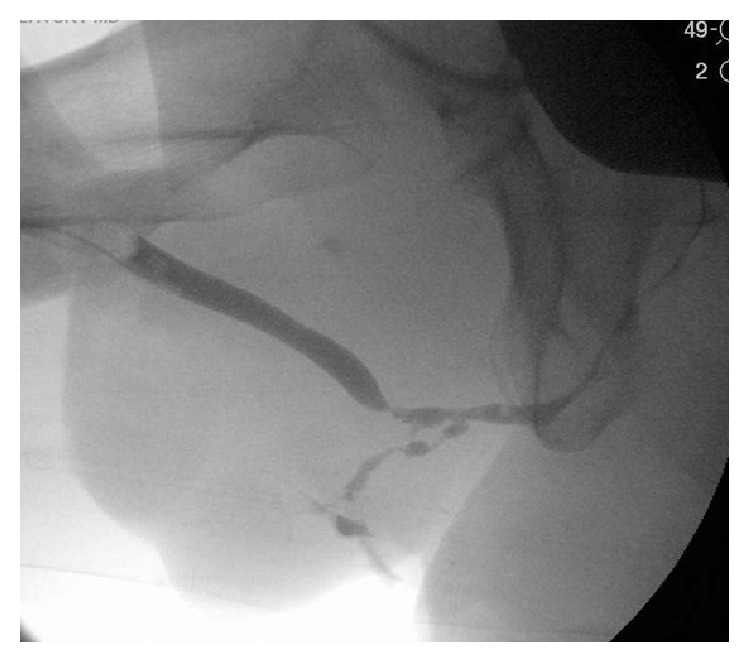
Preoperative retrograde urethrogram and attempted voiding cystogram.

Three months later he presented for reevaluation with a voiding retrograde urethrogram and attempted voiding cystourethrogram (Figure 1). The study demonstrated a 5 cm stricture involving most of the bulbous urethra and a persistent urethrocutaneous fistula draining into the perineum. Patient was counseled on available reconstruction and diversion techniques, and had chosen reconstruction. In view of his prior extensive reconstruction with local flaps and subsequent significant trauma to the perineum followed by abscess a reliable local tissue was not available for reconstruction. The plan was to excise the devastated urethral segment and reconstruct it by augmenting the defect with buccal mucosal graft dorsally and composite buccal mucosa-gracilis flap ventrally. The procedure was planned to be performed in two stages to monitor graft survival and contracture before committing to “tubularization.”

**Figure 2:**
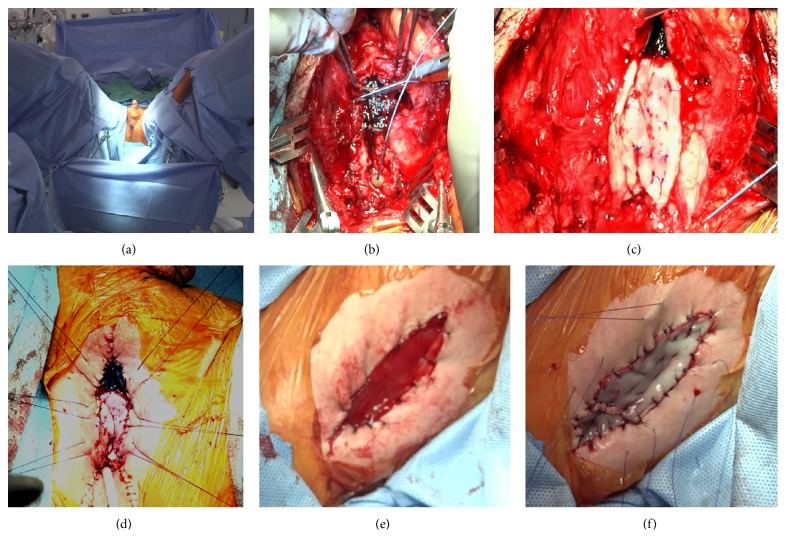
Steps of the Stage 1 procedure. (a) Patient positioned with access to the perineum and the left inner thigh, (b) transection of the affected urethral plate, (c) dorsal plate augmented with buccal mucosa, (d) augmented perineal urethrostomy with catheter in place (preparation for the tie-over dressing), (e) exposed distal gracilis, and (f) buccal mucosa quilted on the gracilis.

*Stage 1*. After appropriate informed consent was obtained, the patient underwent a general anesthesia, administered IV Cefazolin and Gentamycin, placed in a low lithotomy position, prepped, and draped in a manner allowing access to the perineum and the left inner leg (Figure 2(a)). The suprapubic tube was removed and a suprapubic cystoscopy was performed with an attempt to pass a guide wire antegrade through the bladder neck into the urethra. The wire had preferentially passed into the urethrocutaneous fistula.

*(a) Augmented Perineal Urethrostomy*. An inverted Y-incision was carried through the perineum and through the abundant scar. The fistula and the affected urethra were dissected and removed leading to a 6 cm defect (Figure 2(b)). The proximal and distal urethral stumps were spatulated both ventrally and dorsally and their lumens measured at 24 French. Two 6 × 2.5 cm buccal mucosal grafts were harvested, defatted, and fenestrated (Figure 2(c)). One graft was quilted on the corpora cavernosa and anastomosed to the spatulated urethral stumps dorsally using 4-0 polydioxanone suture. A perineal urethrostomy was matured by approximating edges of skin to the edges of neourethral plate (Figure 2(d)). A catheter was placed in the urethrostomy and a tie-over dressing applied over the graft.

*(b) Gracilis Prelamination*. During the same operation, the left gracilis tendon was identified and a 6 × 2.5 cm elliptical skin defect was created just proximal to the tendon (Figure 2(e)). The exposed muscle was sutured to the skin edges as previously described by Zinman [1]. The second harvested buccal graft was quilted on the exposed muscle with 4-0 polydioxanone sutures and a tie-over dressing and an elastic bandage around the leg were applied for one week (Figure 2(f)). The patient had returned home on postoperative day one and was seen in the clinic at 1-2-week intervals for the graft assessments. Both the urethral catheter and the tie-over dressings were removed at one-week follow-up.

**Figure 3:**
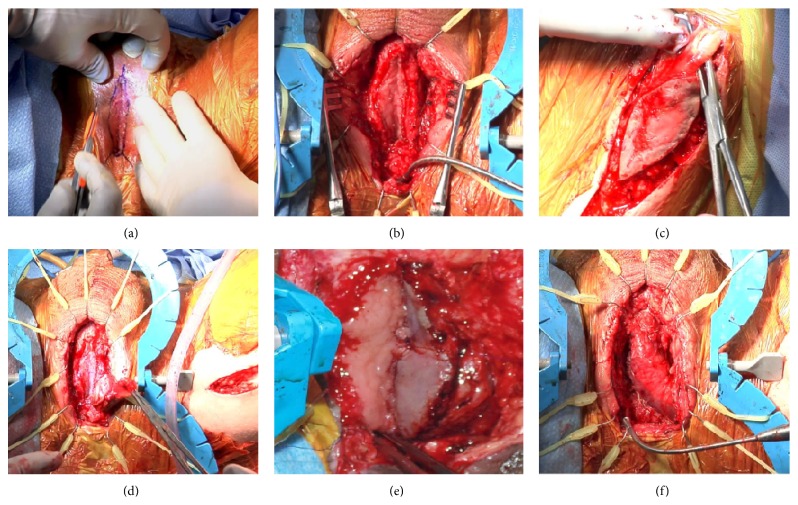
Steps of the Stage 2 procedure. (a) Engrafted perineal urethrostomy at 8 weeks, (b) dorsal plate dissected, (c) gracilis-buccal mucosa composite harvest, (d) gracilis tunneled into the perineal incision, (e) dorsal (left) and ventral (right) plates anastomosed on the left side, and (f) completed anastomosis of ventral and dorsal hemi-plates.

*Stage 2*. Eight weeks after the first stage, both grafts appeared to have taken well with no signs of a contracture or stenosis of the urethrostomy (Figure 3(a)). Again, the patient was placed in a low lithotomy position and prepped with access to the perineum and the left inner thigh. The perineal urethrostomy was freed by incising the skin just around the edges of the buccal graft (new dorsal hemi-plate) and dissecting laterally away from the graft (Figure 3(b)). Both proximal and distal urethral stumps were again calibrated with a bougie at 24 French.

**Figure 4:**
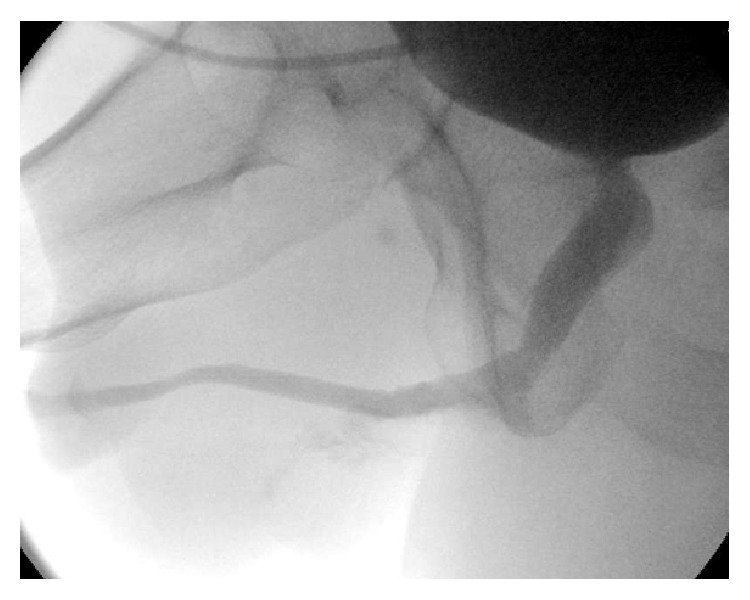
Voiding cystourethrography at 5 weeks.

The second incision was made around the buccal mucosa engrafted on gracilis and extended distally and proximally 3–5 cm along the axis of the muscle. The muscle was circumferentially dissected proximally and distally and its tendon transected (Figure 3(c)). The minor distal vascular pedicles to gracilis were tied and severed. To facilitate the gracilis harvest and its tunneling, another 5 cm incision was made along the course of the muscle equidistant between the perineal incision and the distal gracilis incision. The major proximal vascular pedicle located approximately 10 cm distal to the pubic ramus was preserved. The muscle-graft composite was passed through a wide tunnel into the perineal incision and the BMG edges on the flap (new ventral plate) were approximated apex-to-apex to the edges of the dorsal graft with running 4-0 polydioxanone sutures (Figures 3(d)–3(f)). A 16-French silicone catheter was left in place. The gracilis muscle was sutured to corpora cavernosa as a second layer over the first suture line with a 3-0 polyglactin suture. Both the perineal and the leg incisions were closed in layers and a Jackson-Pratt drain was left in the gracilis harvest bed for one week. The patient returned home one day after the operation and was followed in the office at 1-2-week intervals. He had retrograde and antegrade imaging studies in the office at 3 weeks demonstrating a small contrast extravasation which had resolved by 5 weeks at which point his urethral catheter was removed (Figure 4). The suprapubic catheter was removed 6 weeks later after a period of successful voiding with tube clamped.

### 2.1. Follow-Up

**Figure 5:**
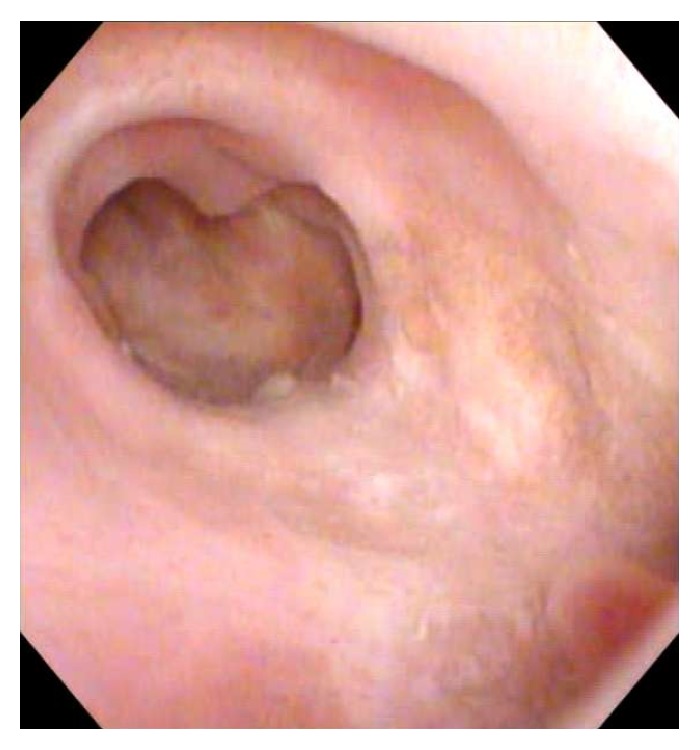
Urethroscopy at 8-month follow-up.

At 8-month follow-up patient was voiding with a maximum flow of 53 cc/sec and a postvoid residual of 51 cc. He has reported no urinary incontinence and no fistulous connection to the perineum itself. He has demonstrated a relatively preserved erectile function with SHIM score decreasing from 21 preoperatively to 18 postoperatively. Through his follow-up patient reported having neither chordee nor pain with erections. As expected, he admitted diminished force of ejaculation (since before his trauma) and minimal postvoid dribbling. On the office cystoscopy at 8-month follow-up the patient demonstrated patent anastomotic sites and a patulous neourethral segment circumferentially lined with healthy appearing buccal mucosa (Figure 5). The patient has reported working without limitations and has not been having any functional disability or changes in his gate.

## 3. Discussion

A necrotic or devastated urethra presents a daunting problem especially in salvage situations where a reliable local tissue is unavailable due to an infection, fistulae, numerous prior failed operations, radiation, or extensive lichen sclerosis. In some situations patients are commonly counseled on benefits of diversion or perineal urethrostomy and “heroic measures” are discouraged [2]. However, for the patients insisting on reconstruction the available techniques are extremely challenging. Enterourethroplasty techniques using various GI segments in 11 patients with otherwise unsalvageable urethras were described by Mundy and Andrich [3]. The benefit of this procedure is a one-stage repair immediately producing tubular urethra. The drawbacks of this procedure are the need for abdominal and perineal approach, and the need for enteroenteric reanastomoses with their predictable potential complications. The resulting colonic or duodenal urethra is then theoretically prone to diverticulae formation and mucus production. This technique could be ideal when a two-stage repair is not accepted by a patient or in situations when a temporary perineal urethrostomy is not technically feasible, or when a long segment of absent bulbar urethra is accompanied by additional obliterated or diseased posterior urethra.

An alternative innovative 3-stage approach was described by Wu et al. [4]. In the first stage the affected bulbous urethra is excised and the pendulous urethral stump is directly anastomosed to the spatulated proximal stump. This maneuver leaves the phallus completely invaginated for 6 months. During the second stage the phallus is dissected from the urethra leaving a hypospadiac meatus as the pendulous urethra now becomes the bulbous urethra. In the final stage the pendulous neourethra is created and tubularized similar to a hypospadias repair. The drawback of this procedure is a need for numerous operations and eventual need for the pendulous urethral tubularization fraught with risks of fistula formation and poor cosmetic appearance. This procedure is suitable in situations where a perineal urethrostomy is not technically feasible, but a patient is willing to undergo a staged procedure.

Another innovative technique was described by Kulkarni et al. and involves an exposed dartos prelamination with buccal mucosa and a subsequent flap-graft tubularization [5]. This technique creates a neourethra circumferentially lined with buccal mucosa but requires availability of preserved scrotal tissue and, after harvest, relies on a random blood supply. The authors have reported on 8 patients treated with this technique and noted a 50% medium-term patency rate and frequent formation of large diverticula.

Several other techniques have been described using preputial or penile fasciocutaneous flaps either being tubularized or in a form of ventral patches in combination with dorsal buccal mucosa onlay [5–7]. The main advantage of these techniques is the ability to create a long urethral segment in one stage. The disadvantages of these techniques are frequent unavailability of the required tissue (i.e., history of circumcision, prior use of penile skin) and common formation of large diverticula when these flaps are used [5].

The development of the technique presented in this case report has relied on modification of several previously published reports of prelamination of gracilis with skin grafts for staged urethral reconstruction [1, 8, 9]. In 2002 Zinman has described a technique used in four patients with devastated urethras which were patched with a split-thickness skin-gracilis composite flap prefabricated 3–6 weeks earlier [1]. The outcomes of the procedure were not described. Camp et al. described gracilis prelamination with full-thickness skin graft for a delayed tubularization and formation of a missing segment of neourethra in a female-to-male transgender patient [8]. The authors used a hairless skin from the forearm for prelamination on gracilis to avoid hair formation within the urethra. No follow-up was reported beyond catheter removal at 3 weeks. Crane et al. had published a case report describing gracilis prelamination with a full-thickness skin graft. Two weeks later this flap-graft was used as a patch to augment portions of damaged male urethra [9]. The authors reported on excellent cosmetic and functional outcomes at 9 months of follow-up.

To our knowledge ours is the first report demonstrating feasibility of using prefabricated buccal mucosa-gracilis composite flap for repair of devastated urethra. This technique allows avoiding the use of hair-bearing skin or recruitment of gastrointestinal segments. A gracilis muscle provides a vascular bed ventrally and reduces the risk of developing a ventral diverticulum or a fistula. Creation of separate ventral and dorsal plates obviates circumferential tubularization and thus avoids excessive mobilization of the engrafted mucosa. Independently vascularized urethral hemi-plates require minimal dissection to achieve a truly tension-free anastomosis. A larger multi-institutional study is being conducted to evaluate long-term outcomes of this technique.
